# Single‐center study of autoimmune encephalitis‐related autoantibody testing in Hungary

**DOI:** 10.1002/brb3.1454

**Published:** 2019-10-24

**Authors:** Zsófia Hayden, Katalin Böröcz, Zsuzsanna Csizmadia, Péter Balogh, Zoltán Kellermayer, Kornélia Bodó, József Najbauer, Tímea Berki

**Affiliations:** ^1^ Department of Immunology and Biotechnology Clinical Center University of Pécs Medical School Pécs Hungary

**Keywords:** autoantibodies, autoimmune encephalitis, biochip, laboratory diagnostics

## Abstract

**Objective:**

Autoantibody detection is crucial for the early diagnosis of autoimmune encephalitis (AIE) since prompt therapy can determine the disease outcome. Here, we report a single‐center 6‐year retrospective study of autoantibody testing in AIE in the Hungarian population.

**Methods:**

Serum and/or cerebrospinal fluid (CSF) autoantibody tests were performed using cell‐based indirect immunofluorescence assay for AIE diagnosis. Samples were provided by neurology clinics as part of a nationwide program. Test results were analyzed for samples received during the period from 2012 to 2018.

**Results:**

We tested 1,247 samples from 1,034 patients with suspected AIE. Autoantibodies were present in 60 patients (5.8% of total). The distribution of patients with different autoantibodies by age and sex was as follows: NMDAR (70%), mostly in young females, LGI1 (15%) in middle‐aged males, GABA_B_R (12%) in elderly males, and Caspr2 (7%) in males. Long‐term follow‐up was conducted in 30 patients with repeated test requests, of which 17 remained positive, and 13 switched to negative.

**Conclusion:**

We report the most comprehensive clinical laboratory study of autoantibody testing in AIE in the Hungarian population. Our results show that the frequency of different autoantibody types in AIE corresponds to the data described in the literature.

## Graphical abstract

We report the most comprehensive clinical laboratory study of autoantibody testing in AIE in the Hungarian population. Our results show that the frequency of different autoantibody types in AIE corresponds to the data described in the literature. In agreement with the international recommendations, our data support that for AIE diagnosis both serum and CSF samples should be tested.

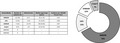

## 1 INTRODUCTION

During the past few years, it has been recognized that there are central nervous system (CNS) disorders presenting in the form of limbic encephalitis, in which the presence of autoantibodies against the neuronal cell surface receptors such as NMDAR, GABA_B_R, and AMPAR or synaptic proteins, LGI1 and Caspr2, has been documented and shown to be responsible for the development of the symptoms (Dalmau, Geis, & Graus, 2017; Dalmau & Graus, 2018; Newman et al., 2016). The target molecules of these autoantibodies play important roles in synaptic signal transmission and neuronal plasticity. The autoimmune reaction to these antigens in the majority of cases leads to epileptic seizures and neuropsychiatric symptoms (Table 1) (Celicanin et al., 2017; Fukata, Yokoi, & Fukata, 2018; Honnorat & Plazat, 2018; van Sonderen, Petit‐Pedrol, Dalmau, & Titulaer, 2017; Szots et al., 2017). In autoimmune encephalitis (AIE), the autoantibodies bind to the extracellular epitopes of the neuronal cell surface receptors or their associated proteins, which can lead to alteration of the structure and function of target antigens by different mechanisms. Thus in anti‐NMDAR encephalitis, autoantibodies induce receptor cross‐linking and internalization, in anti‐LGI1 encephalitis autoantibodies interfere with protein–protein interactions, and in anti‐GABA_B_R encephalitis autoantibodies may block the function of the target antigen (Hughes et al., 2010; Ohkawa et al., 2013). The autoantibodies cause reversible neuronal dysfunction, and immunotherapy (e.g., steroids, plasmapheresis, immunosuppression, and intravenous immunoglobulin) results in reduction of autoantibody levels and can lead to the improvement of patients (Hermetter, Fazekas, & Hochmeister, 2018). Patients can have a fatal outcome in case of lack of the proper therapy. This highlights the importance of early clinical diagnosis of AIE, in which the laboratory has a crucial role by providing accurate and reproducible testing of serum and/or cerebrospinal fluid (CSF) samples for the presence of autoantibodies.

**Table 1:** Main characteristics of different autoimmune encephalitis types

Autoantibody	Clinical features	MRI (T2/FLAIR)	Tumor	Prognosis	Male/Female	Median age (years)
NMDAR	Prodromal stage (fever, headache, abdominal pain) Psychiatric symptoms (agitation, hallucinations, delusions, catatonia, psychosis) Later manifestations (reduction of speech, memory deficit, orofacial and limb dyskinesias, seizures, decreased level of consciousness, autonomic instability)	Normal or nonspecific changes	58%, (age‐ and sex‐dependent) in young women ovarian teratoma	81% have a good outcome	1:4	21
LGI1	Faciobrachial dystonic seizures, limbic encephalitis, hyponatremia, sleep disorders, memory, and cognitive deficits	Hyperintense signal in medial temporal lobes	˂5%, thymoma	70% have a good outcome	2:1	64
Caspr2	Neuromyotonia, Morvan's syndrome, limbic encephalitis, insomnia, neuropathic pain	Hyperintense signal in medial temporal lobes	˂5%, thymoma	70% have a good outcome	9:1	66
GABA_B_R	Limbic encephalitis, seizures Rarely: cerebellar ataxia, opsoclonus‐myoclonus	Hyperintense signal in medial temporal lobes	50%, SCLC	80% initially good response but have poor prognosis due to SCLC	1.5:1	61
AMPAR	Limbic encephalitis, seizures Rarely: psychiatric symptoms	Hyperintense signal in medial temporal lobes	56%, SCLC, thymoma, or breast carcinoma	70% have a good outcome	1:2.3	56

Note

Based on Dalmau and Graus (2018), Newman et al. ( 2016), van Sonderen et al. (2017).

## 2 MATERIALS AND METHODS

### 2.1 Samples

We carried out a retrospective statistical study of the results obtained by our laboratory based on serum and CSF analysis of patients with suspected AIE. Our laboratory was the first to introduce these tests in Hungary and has received samples from various neurological clinics and hospitals from 2012 through 2018 as part of a nationwide program. Serum and CSF samples were obtained with patients' informed consent. The study was approved by Regional Research Ethics Committee of the Medical Center, University of Pécs (RIKEB 6966/2017).

### 2.2 Detection of AIE autoantibodies

For detection of AIE‐related autoantibodies, a cell‐based indirect immunofluorescence BIOCHIP assay was used (Euroimmun, Autoimmune Encephalitis Mosaic 1, FA 112d‐1003‐1). On the BIOCHIP slide, HEK293 cells expressing six different antigens of interest (NMDAR, LGI1, Caspr2, GABA_B_R, AMPAR1, and AMPAR2) are immobilized as a mosaic. Five samples can be investigated on a single slide; one mosaic is suitable for the detection of six types of autoantibodies. Optimization of the assay was based on the recommended protocol included in the Manufacturer's Instruction. About 30 µl of the samples (sera diluted 1:10 or CSF undiluted) were incubated on the BIOCHIP containing the six transfected cell lines for 30 min at room temperature, followed by two washing steps with PBS Tween‐20 buffer (included in the kit) for 5 min. For secondary labeling, 25 µl of anti‐Human IgG (Fc‐specific)‐FITC antibody specifically recognizing Fc fragment of all human IgG subclasses (IgG is the most frequently associated immunoglobulin isotype in AIE (Ricken et al., 2018)), included in the kit, was applied for 30 min at room temperature. After two washes for 5 min, glycerol (included in the kit) was used for covering the slides. Positive and negative controls were used to help evaluate the patient samples. The advantage of this test is the capacity of detecting simultaneously the presence of six different types of autoantibodies in a single sample. In case of positive or equivocal test results for anti‐NMDAR autoantibodies, the anti‐Glutamate Receptor (type NMDA) IIFT kit was used as a confirmatory test (Euroimmune). These BIOCHIP slides contain NMDAR expressing and control HEK293 cells immobilized as a mosaic. Optimization was also based on the Manufacturer's Instruction.

### 2.3 Fluorescence imaging and evaluation

Fluoresce imaging was performed using a fluorescence microscope (Olympus BX61) coupled with Zeiss Axiocam 305 color microscope digital camera and image processing system. The BIOCHIPs were evaluated independently by at least two laboratory specialists. Positive and negative controls were used, and reactions were graded as strong positive, positive, low positive, equivocal, and negative.

## 3 RESULTS

### 3.1 Annual distribution and frequency of autoimmune encephalitis‐related autoantibody types

**Figure 1:**
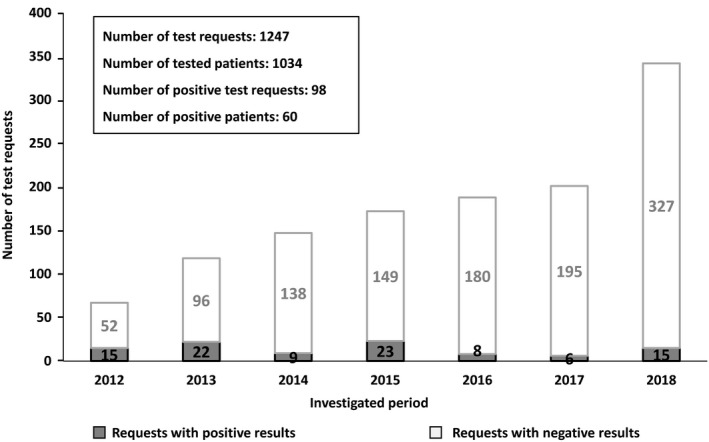
Annual distribution of autoimmune encephalitis test requests. The cell‐based indirect immunofluorescence BIOCHIP assay was introduced at our laboratory in 2012, and test results were analyzed through 2018. The number of positive tests varied in time, although the total number of requests increased each year. Data of 12 equivocal test results are not shown

**Figure 2:**
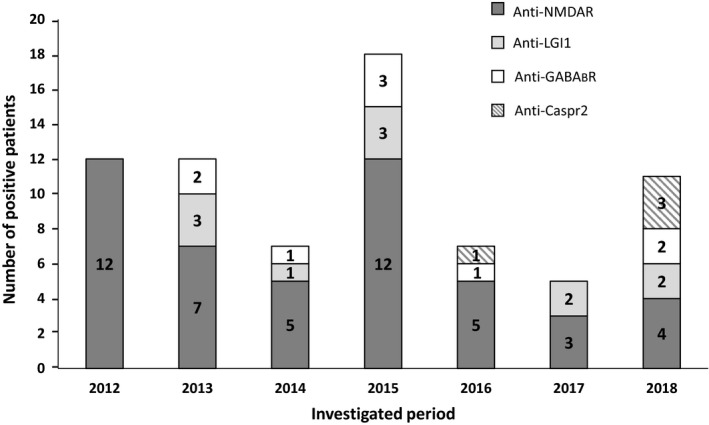
Annual distribution of the positively detected autoantibody types in autoimmune encephalitis patients. The ratio of autoantibody‐positive patients in decreasing order was NMDAR > LGI1 > GABA_B_R > Caspr2. No patients were found with anti‐AMPAR1 or anti‐AMPAR2 autoantibody positivity

**Figure 3:**
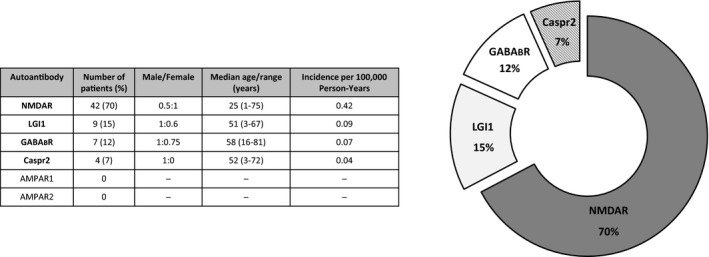
Frequency and distribution of autoantibody types by age and sex in autoimmune encephalitis patients. The inserted table shows the number of patients belonging to the different AIE autoantibody types. Anti‐NMDAR autoantibodies were most frequently detected in young women, anti‐LGI1 autoantibodies occurred in middle‐aged males, anti‐GABA_B_R autoantibodies were present in elderly males, and anti‐Caspr2 autoantibodies affected adult males

Since the introduction of tests for AIE in 2012 at our institution, the number of test requests for diagnosing the disease has increased each year. Our laboratory has received 1,247 test requests (sera and/or CSF samples) from a total of 1,034 patients for detection of AIE‐related autoantibodies (Figure 1). We employed a cell‐based indirect immunofluorescence BIOCHIP assay for the detection of six ion channel or their associated protein‐specific autoantibodies (NMDAR, Caspr2, GABA_B_R, AMPAR1, AMPAR2, and LGI1). We have found 98 positive samples belonging to 60 patients. This result reflects that autoantibodies were present in only 5.8% of the patients with clinically suspected AIE. The frequency of the positive samples varied with the examination period; the highest ratio of positive test requests was during the first 4 years after the introduction of the test [22.4% (2012), 18.7% (2013), 6.1% (2014), 13.4% (2015)]. Although the number of test requests continued to increase, the ratio of positivity was lower during the past 3 years [4.3% (2016), 3% (2017), 4.4% (2018)]. The highest proportion of positive tests for AIE was 22.4% (2012), and the lowest was 3% (2017) (Figure 1). By analyzing the annual distribution of patients with positive AIE test results, we found marked differences. The number of newly diagnosed patients was 3–14/year, while requests with positive results of the already diagnosed positive patients were only 1–4/year (data not shown). Figure 2 shows the annual distribution of positive patients with different types of autoantibodies. Patients with anti‐NMDAR antibodies showed the highest frequency for each year during the examined period. The frequency of different types of autoantibodies also varied: anti‐NMDAR autoantibody was present in 70%, anti‐LGI1 in 15%, anti‐GABA_B_R in 12%, and anti‐Caspr2 in 7% of patients (Figure 3). Two patients showed positivity for two types of autoantibodies simultaneously (one patient showed positivity against LGI1 and Caspr2, and the other against GABA_B_R and Caspr2). In 12 patients, the results obtained from sera were equivocal (11 NMDAR and one GABA_B_R); of which, five patients were negative upon simultaneous testing of CSF, and new samples from three patients were negative upon retesting.

### 3.2 Characteristics of patients with positive autoantibodies

We investigated the distribution of AIE‐related autoantibody subtypes by age and sex (Figure 3). Anti‐NMDAR encephalitis mostly affected females: of the 42 anti‐NMDAR‐positive patients, 28 were female, with median age of 25 years. Anti‐LGI1 encephalitis most frequently occurred in middle‐aged males: of the nine anti‐LGI1‐positive patients, six were male, with median age of 51 years. Anti‐GABA_B_R encephalitis affected elderly males: of the seven anti‐GABA_B_R‐positive patients, four were male, with median age of 58 years. Anti‐Caspr2 encephalitis occurred in male adults: of the four anti‐Caspr2‐positive patients, three were male, with median age of 52 years (Figure 3). Two patients showed positivity for two types of autoantibodies simultaneously: a 3‐year‐old boy was positive for both anti‐LGI1 and anti‐Caspr2, and a 60‐year‐old male was positive for anti‐GABA_B_R and anti‐Caspr2. Of the total 60 positive patients, in 30 cases repeated tests were performed at different time points (Table 2). In 17 cases, repeated laboratory tests resulted in autoantibody positivity multiple times, while in 13 patients the original positive autoantibody result subsequently switched to negative. Five patients (one anti‐NMDAR, one anti‐LGI1, one anti‐GABA_B_R, and one positive for both anti‐LGI1 and anti‐Caspr2 antibodies) became negative within 1 year after the first positive test; four anti‐NMDAR‐positive patients switched to negative within the 2nd year after the first positive test; three patients (two anti‐NMDAR and one anti‐LGI1) turned to negative within the 3rd year; and one anti‐NMDAR patient was found negative during the 5th year after the first positive test (Table 2).

**Table 2:** Groups of autoantibody‐positive patients with repeated test requests

Patients	ID	Autoantibody type	Follow‐up time (years)	Number of test requests
Repeatedly positive	1.	NMDAR	1 (2013)	7
	2.		5 (2014–2018)	3
	3.		2 (2013–2014)	2
	4.		1 (2015)	2
	5.		2 (2015–2016)	2
	6.		1 (2012)	2
	7.		1 (2012)	2
	8.		4 (2012–2015)	2
	9.		1 (2018)	2
	10.		1 (2015)	2
	11.		2 (2016–2017)	2
	12.		1 (2015)	2
	13.		1 (2015)	2
	14.	LGI1	3 (2013–2015)	4
	15.	GABA_B_R	3 (2013–2015)	3
	16.		1 (2015)	2
	17.		1 (2018)	2
Switched to negative	1.	NMDAR	5 (2012–2016)	7
	2.		3 (2016–2018)	4
	3.		2 (2012–2013)	2
	4.		1 (2017)	2
	5.		2 (2012–2013)	2
	6.		2 (2012–2013)	2
	7.		2 (2012–2013)	2
	8.		3 (2012–2014)	2
	9.	LGI1	1 (2013)	2
	10.		1 (2018)	2
	11.		3 (2013–2015)	3
	12.	LGI1 + Caspr2	1 (2018)	3
	13.	GABA_B_R	1 (2015)	2

### 3.3 Influence of sample type on the laboratory test results

Among the 60 positive patients, in 34 cases (57% of autoantibody‐positive patients) autoantibodies were detected only in serum or only in CSF samples. In 28 cases (47%) only serum, while in six cases (10%) only CSF was tested. In 26 cases (43%), both sample types were investigated. Analyzing in more detail this group of autoantibody‐positive patients, we found marked differences regarding the sample type and the strength of detected positivity in the different AIE‐related autoantibody types. Among 17 anti‐NMDAR patients—whose sera and CSF were tested simultaneously—in seven cases autoantibodies were detected in both serum and CSF, but in three cases stronger positivity was detected in the CSF. In seven anti‐NMDAR patients, positivity was found only in CSF and in three cases only in serum. In three anti‐LGI1 patients, we detected autoantibodies in sera only, but not in the CSF. In four anti‐GABA_B_R patients, positivity was detected in both serum and CSF, and in one case, serum showed higher level of antibodies than CSF. In one anti‐Caspr2 patient, only the serum was positive. In one patient, both anti‐LGI1 and anti‐Caspr2 antibodies were detected in both sample types, although the anti‐Caspr2 positivity was stronger in the serum than in CSF (Table 3).

**Table 3:** Summary of test results of autoimmune encephalitis‐related autoantibodies in patients with serum and/or CSF positivity according to AIE subtypes

Patient ID	Serum	CSF	Autoantibody subtype
1.	+	NA	NMDAR
2.	+	+	
3.	+	NA	
4.	NA	+	
5.	+	NA	
6.	+	NA	
7.	+	NA	
8.	+	NA	
9.	+	NA	
10.	+	NA	
11.	+	+	
12.	+	NA	
13.	+	+	
14.	+	NA	
15.	+	NA	
16.	+	NA	
17.	+	+	
18.	+	+++	
19.	+	NA	
20.	−	+	
21.	NA	+	
22.	−	+	
23.	+	+++	
24.	−	+	
25.	+	NA	
26.	+/−	+	
27.	−	+	
28.	+	NA	
29.	−	+	
30.	NA	+	
31.	NA	+++	
32.	NA	+	
33.	+	++	
34.	+	NA	
35.	++	NA	
36.	NA	+	
37.	+	−	
38.	+	NA	
39.	+	−	
40.	−	++	
41.	+	−	
42.	++	NA	
43.	+	−	LGI1
44.	+	NA	
45.	+	NA	
46.	+	NA	
47.	++	NA	
48.	+	−	
49.	+++	NA	
50.	++	−	
51.	+	+	LGI1 and Caspr2
	++	+	
52.	+	+	GABA_B_R
53.	+	+	
54.	+	+	
55.	+	NA	
56.	++	NA	
57.	++	+	
58.	+	NA	GABA_B_R and Caspr2
	+	NA	
59.	+	NA	Caspr2
60.	+	−	

Abbreviations: −, negative; +, low positive; +/−, equivocal; ++, positive; +++, strong positive; NA, not available.

## 4 DISCUSSION

AIE has been recognized during the past decade as a distinct disease entity (Dalmau & Graus, 2018; Venkatesan, Michael, Probasco, Geocadin, & Solomon, 2019). The discovery of AIE subtypes has changed the diagnostic and therapeutic approaches to many neurological disorders previously considered to be idiopathic. Our aim was to investigate the characteristics of autoantibody testing in patients with AIE, in which early and accurate clinical diagnosis plays a pivotal role. The current retrospective analysis included laboratory test results from 1,034 patients with suspected AIE, making it the most comprehensive study in Hungary to date. Our data confirmed the relative prevalence of AIE subtypes described previously (Dalmau & Graus, 2018). Anti‐NMDAR encephalitis was the most common subtype, followed by anti‐LGI1, anti‐GABA_B_R, and anti‐Caspr2 encephalitis, which is in agreement with previous reports (Gable, Sheriff, Dalmau, Tilley, & Glaser, 2012; van Sonderen et al., 2017). Our data regarding the age and sex of AIE patients agree with the data published in the literature (Ricken et al., 2018).

Highly sensitive and specific multiplex cell‐based assay is available for AIE diagnostics, in which HEK293 cells expressing high levels of antigens of interest are used. Analysis of serum and CSF samples of patients with AIE suggested that both types of samples should be tested, especially in patients with anti‐NMDAR autoantibodies, since in most patients the autoantibodies are detected in the CSF, while the serum might be negative. However, in some anti‐LGI1 antibody‐positive patients, autoantibodies can be found only in serum or only in CSF (Table 3) (Dalmau & Graus, 2018). It has been reported previously that detection of the characteristic autoantibodies in clinically suspected AIE could serve as confirmatory diagnosis, and in case of anti‐NMDAR encephalitis, testing of CSF can be used to monitor disease activity, and autoantibody levels often correlate with patient outcome and relapse rates (Lee & Lee, 2016; Wandinger, Leypoldt, & Junker, 2018). The highest sensitivity and specificity of the tests can be achieved by testing both serum and CSF. It should be noted, however, that false‐positive results can occur more commonly when serum samples are tested.

## 5 CONCLUSION

In conclusion, our data are in agreement with previous reports on the frequency and distribution of AIE‐related autoantibodies, and detection of which can significantly aid the diagnosis of AIE and suggests treatment strategies. Early immunotherapy is often effective and can reduce the severity of AIE, promote recovery and decrease the risk of relapse (Crisp, Kullmann, & Vincent, 2016; Dalmau & Graus, 2018; Ricken et al., 2018; Varley, Taylor, & Irani, 2018). As the number of patients affected by AIE is increasing and the spectrum of the newly identified autoantibodies broadens, it is important to employ reliable laboratory tests that allow accurate diagnosis to be made. The evaluation of patients with suspected autoimmune encephalitis should include testing for autoantibodies in both serum and CSF simultaneously, since some autoantibodies can be preferentially found only in serum or in CSF (Dalmau & Graus, 2018). Finally, early recognition of AIE subtypes is important because without proper treatment they can have fatal outcome.

## CONFLICT OF INTEREST

The authors declare that the research was conducted in the absence of any commercial or financial relationships that could be construed as a potential conflict of interest.

## AUTHOR CONTRIBUTIONS

ZH, KaB, ZC, PB, ZK, and TB performed the tests and evaluated the results. ZH, KaB, and TB analyzed data. ZH and KoB performed imaging of indirect immunofluorescence staining of BIOCHIP using an Olympus BX61 fluorescence microscope. ZH, JN, and TB wrote the paper with editorial help from PB and ZK.

## Data Availability

The data that support the findings of this study are available from the corresponding author upon reasonable request.
